# P23 Procalcitonin testing in acute infection to guide antibiotic course lengths in the ICU as a tool for antibiotic stewardship

**DOI:** 10.1093/jacamr/dlaf118.030

**Published:** 2025-07-14

**Authors:** Miriam Beattie, James Donachie, Laura Stewart, Simon Gill, David Cain, Neil Powell

**Affiliations:** Critical Care, Royal Cornwall Hospital, Truro, TR1 3LJ, UK; Critical Care, Royal Cornwall Hospital, Truro, TR1 3LJ, UK; Pharmacy Department, Royal Cornwall Hospital, Truro, TR1 3LJ, UK; Critical Care, Royal Cornwall Hospital, Truro, TR1 3LJ, UK; Critical Care, Royal Cornwall Hospital, Truro, TR1 3LJ, UK; Pharmacy Department, Royal Cornwall Hospital, Truro, TR1 3LJ, UK

## Abstract

**Background:**

Procalcitonin (PCT) was introduced into the ICU during the COVID-19 pandemic. PCT enables optimization of antibiotic course lengths for ICU patients, positively impacting antimicrobial stewardship.

**Objectives:**

To evaluate local adherence to PCT testing for patients with suspected acute bacterial infections in an ICU in England.

**Methods:**

Patients discharged from the ICU between 1 November and 30 November 2023 were identified. PCT test timing, frequency, PCT level, antibiotic course lengths (both in the ICU and after ICU discharge) and infection diagnosis were extracted. Adherence to a proposed local PCT testing protocol was determined and the medical notes reviewed for evidence of PCT guided antibiotic decision making.

**Results:**

Of 65 patients discharged from ICU, 49 (75.4%) received antibiotics during their ICU stay. Thirty-six (77.6%) of these patients had 40 antibiotic courses for suspected acute bacterial infections. A baseline PCT was ordered in 18/40 (45.0%), a repeat PCT ordered in 6 (46.2%) antibiotic courses. The median antibiotic course length for acute infection in the ICU was 2.98 days (IQR 1.72–4.73), with little evidence of PCT-guided antibiotic decision making.

**Conclusions:**

Within our ICU, PCT measurement is sporadic and often not in accordance with the proposed local PCT-testing algorithm. Antibiotic course lengths, both during the ICU stay and total antibiotic courses, were short.

